# Editorial: Towards targeting prolactin signaling in human diseases: stimulate or inhibit?

**DOI:** 10.3389/fendo.2023.1213895

**Published:** 2023-06-29

**Authors:** Vincent Goffin, Damasia Becu-Villalobos, Vera Popovic, David R. Grattan

**Affiliations:** ^1^ Université Paris Cité, Paris, Île-de-France, France; ^2^ PRL/GH Pathophysiology, Institut National de la Santé et de la Recherche Médicale (INSERM) U1151 Institut Necker Enfants Malades, Paris, Île-de-France, France; ^3^ Laboratory of Pituitary Regulation, Consejo Nacional de Investigaciones Cientificas y Tecnicas (CONICET) Institute of Biology and Experimental Medicine (IBYME), Buenos Aires, Argentina; ^4^ Faculty of Medicine, University of Belgrade, Belgrade, Serbia; ^5^ Centre for Neuroendocrinology, Division of Health Sciences, University of Otago, Dunedin, Otago, New Zealand

**Keywords:** prolactin, disease, cancer, metabolism, inflammation, pituitary, behaviour, therapy

Prolactin is an anterior pituitary hormone that was originally named for its indispensable role in lactation, but increasingly it is being recognized for pleiotropic roles in metabolism, immune function, pregnancy adaptations and parental behavior. Prolactin secretion is tightly controlled by a short-loop feedback system whereby prolactin stimulates specific neurons in the hypothalamus to release dopamine, which then inhibits prolactin secretion. During pregnancy and lactation, however, this feedback systems adapts to allow prolonged elevations in prolactin secretion, enabling a range of functions specific to these conditions. Prolactin is also released under conditions of stress in both sexes. Prolactin signals exclusively through the prolactin receptor (Prlr), but this is not a simple system. In target cells, prolactin/Prlr engages various signal transduction mechanisms including JAK2/STAT5 (canonical), PI3K/Akt, MAPK and Src family kinases. There is also evidence of local production of prolactin in non-pituitary tissues, leading to autocrine/paracrine receptor triggering independent of circulating hormone. Adding to this complexity, in many species, including humans, there are multiple ligands for the Prlr. These include placental lactogens that supplement prolactin function in pregnancy, and in primates only, pituitary growth hormone. Moreover, specific proteolytic products of these hormones exert important biological actions independent of Prlr. These functions, that are often completely distinct from those of prolactin, have led to the classification of these fragments as a new class of hormones known as vasoinhibins.

Reflecting this molecular and functional complexity, abnormalities in prolactin signaling have been implicated in multiple clinical conditions. There are consequences when circulating prolactin is too high, with hyperprolactinemia causing infertility in both males and females, as well as being associated with a range of metabolic disturbances and mood disorders. But there are also consequences if prolactin is too low, the most obvious being lactation failure in the absence of prolactin signaling, but many other more subtle deficits are being identified. Changes in autocrine/paracrine prolactin signaling may be extremely important in some conditions, e.g. in modulating inflammation, pain responses, and cancer. The challenge remains when to stimulate and when to inhibit prolactin actions, and this Research Topic dissects different situations that would benefit from either option.

Kavarthapu and Dufau provide an integrated view of the molecular biology of the “target” (Prlr) encompassing its complex transcriptional regulation *via* multiple promoters, the various receptor isoforms resulting from alternative splicing, their specific signaling capacities when homo- or hetero-dimerized, their crosstalk with EGFR/HER2 family members, and how these individual processes can cooperatively promote breast cancer progression. In line with this, Schuler and O’Leary provide a systematic overview of the epidemiological and experimental data documenting the complex actions of prolactin in breast cancer, dichotomizing effects on early lesions *versus* established tumors, and showing how the stromal environment (including matrix stiffness) may alter the responses of target cells to prolactin. This balanced perspective tentatively links to the viewpoint of Ali et al. supporting the beneficial actions of prolactin as a pro-differentiation pathway restricting breast cancer cell plasticity, following emerging evidence that preventing epithelial-to-mesenchymal transition and acquisition of stemness may be a viable approach to temper cancer progression.

This Research Topic also highlights the involvement of prolactin in two diseases besides cancer. Triebel et al. provide an up-to-date discussion of the anti-angiogenic and anti-vasopermeability properties of prolactin and vasoinhibins, which may help restrict the vascularization in the eye of patients with diabetes. The translational potential is advocated by results of a clinical trial in which higher prolactin levels were associated with less diabetic retinopathy.

Prolactinomas are the most frequent functional pituitary tumors causing systemic hyperprolactinemia, with its clinical consequences, and mass effect, locally. The first-line treatment involves dopamine receptor D2 agonists, but a minority of patients with prolactinomas are resistant to this therapy. Ferraris explores impaired autocrine actions of prolactin (a local inhibitor of lactotroph proliferation acting through Prlr), independent of dopamine, in a subtype of medically-resistant prolactinomas. This implies that the pathogenesis of these prolactinomas is not the same as those responding to dopamine therapy, raising Prlr-targeting as a potential therapeutic approach.

Three additional papers emphasize the role of prolactin as a homeostasis hormone:

Macotela et al. discuss evidence for the beneficial actions of functional hyperprolactinemia (moderately-elevated prolactin levels) in metabolic diseases such as obesity and non-alcoholic fatty-liver disease. In clinical practice, beyond the role of prolactin in reproduction, a grey zone of hyperprolactinemia is defined as mild to moderate transient elevations of prolactin levels that are poorly understood. Thus, this review defines ‘Homeo Fit-PRL levels’ that are required to deal with metabolic challenges. Clapp et al. describe the roles of prolactin in inflammatory responses, and specifically in rheumatoid arthritis where prolactin has both negative and positive outcomes depending on circulating levels. Inflamed tissue is rich in enzymes that cleave prolactin into the bioactive metabolite vasoinhibins. This hormone also influences tissue responses to inflammation, and thus, when prolactin is high, such as during pregnancy and lactation, levels of vasoinhibins are also increased, providing both direct and indirect mechanisms to influence tissue responses. Finally, Garay et al. focus on placental lactogens, which are pregnancy-specific hormones that act through the Prlr. These hormones are critical to adaptive changes in the mother during pregnancy, and low placental lactogen has been associated with impaired pregnancy outcomes. The authors found that maintaining a conscious healthy diet during pregnancy was associated with increased placental lactogen and increased birthweight of babies.

Should Prlr signaling need to be inhibited in cancer or any other disease, Standing et al. provide the most up-to-date toolbox of the various approaches and drug candidates that have been developed so far, including prolactin-based receptor antagonists, receptor neutralizing antibodies and small molecule inhibitors.

In conclusion, this Research Topic aimed to demonstrate how deciphering the complexity of the prolactin/Prlr system in different tissues has clinical relevance in understanding disease. Collectively, these reviews highlight that for normal function, prolactin must be in the “goldilocks zone” – not too high, and not too low – “just right”. This evidence constitutes a challenge for any therapeutic intervention aimed at modulating systemic Prlr signaling in disease.

## Author contributions

All authors listed have made a substantial, direct, and intellectual contribution to the work and approved it for publication.

